# The Effect of Edge Definition of Complex Networks on Protein Structure Identification

**DOI:** 10.1155/2013/365410

**Published:** 2013-02-28

**Authors:** Jing Sun, Runyu Jing, Di Wu, Tuanfei Zhu, Menglong Li, Yizhou Li

**Affiliations:** ^1^College of Chemistry, Sichuan University, Chengdu 610064, China; ^2^College of Computer Science, Sichuan University, Chengdu 610064, China

## Abstract

The main objective of this study is to explore the contribution of complex network together with its different definitions of vertexes and edges to describe the structure of proteins. Protein folds into a specific conformation for its function depending on interactions between residues. Consequently, in many studies, a protein structure was treated as a complex system comprised of individual components residues, and edges were interactions between residues. What is the proper time for representing a protein structure as a network? To confirm the effect of different definitions of vertexes and edges in constructing the amino acid interaction networks, protein domains and the structural unit of proteins were described using this method. The identification performance of 2847 proteins with domain/domains proved that the structure of proteins was described well when *R*_C_*α*__ was around 5.0–7.5 Å, and the optimal cutoff value for constructing the protein structure networks was 5.0 Å (C_*α*_-C_*α*_ distances) while the ideal community division method was community structure detection based on edge betweenness in this study.

## 1. Introduction

Protein structure comparison and classification are a difficult but important task since structure is a determinant for molecular interaction and function [1]. Protein folds into a specific conformation for its function depending on interactions between residues. Consequently, a protein structure can be treated as a complex system comprised of individual components residues. The method of complex network has been widely applied in various types of fields such as disease [2–4], drug target [5], drug design [6]. Network analysis facilitates the characterization of such complex system and its individual components [7, 8]. This provides novel insights into understanding the protein folding mechanism [9, 10], stability [11], function [9, 12, 13], and dynamics [14] and, more specifically, the study of protein structures. Viewing the protein structure as the an intricate network of interacting residues, metastructure analysis was proved to be an effective tool for large-scale (genome-wide) protein sequence analysis target selection for structural genomics and the identification of intrinsically unstructured (unfolded) proteins [15]. Analysis of the protein structure graphs showed that the aromatic residues along with arginine, histidine, and methionine act as strong hubs at high interaction cutoffs, which are found to play a role in bringing together different secondary structural elements in the tertiary structure of the proteins [11]. Through transforming the protein structure into residue interaction graphs, active site, ligand-binding, and evolutionary conserved residues were found to have high closeness values typically. This property will then be used to identify key protein residues [16]. Moreover, software tools were presented for the automatized generation, 2D visualization, and interactive analysis of residue interaction networks, which proved that residue networks are crucial for understanding structure-function relationships [17]. A novel web server, RING, was presented to construct physicochemically valid residue interaction networks interactively from PDB files for subsequent visualization in the Cytoscape platform [18]. The application of Cytoscape plug-ins, NetworkAnalyzer [19], and RINalyzer [17] were demonstrated for the standard and advanced analyses of network topologies [20].

In these studies, different strategies were used to define a vertex in literature: (a) only the C_*α*_ [9, 10, 15, 21–23] or C_*β*_ [21, 24] of an amino acid; (b) the center of the side chain [11]; (c) all atoms in a residue were taken into account [16, 25]. Moreover, definition of edge also appears crucial in the construction of such networks. The characterization of protein structure is sensitive to the threshold for edges such as 5 Å (distances between two atoms from two amino acid residues) [25], 8 Å (C_*α*_-C_*α*_ distances) [15], 8.5 Å (pairs of amino acids) [9], and a strict cutoff value of 7 Å [9, 10, 15, 21–23] based on the discovery that representing amino acids by C_*α*_ atoms may introduce bias for cutoffs below 6.8 Å [23].

Which strategy is more reasonable among all these choices? Studies have been made to find the answer. Three models were compared to prove the effects of the anisotropic nature of the side chain on the identification of the contact amino acid pairs [26]. The main objective of this study is to explore the contribution of complex network together with its different definitions of vertexes and edges to describing the structure of proteins. Automatic decomposition of protein structures into domains remains a challenging problem [27], and numbers of computer algorithms have been proposed [27–30]. Since domains can be considered as semi-independent structural units of a protein capable of folding independently [31, 32], consequently, the identification of protein domains is an efficient way to present whether a method can describe the protein structure well. In addition, the connections between the residues are dense within these structural units, which are similar to the connections between communities of the complex networks, expressing the community properties of such network well. To facilitate the understanding of such complex systems, community division was used to analyze these amino acid interaction networks. The purpose of this method is to divide the vertexes of the networks into groups, within which the connections between the vertexes are dense and the connections between which are sparser in the same time [33]. Moreover, a number of the methods based on community have been published in many fields [34–39].

In this study, protein structures were represented by complex networks, in which a vertex is a residue and an edge is an interaction between residues. Here, different cutoff values and strategies used for defining a vertex were tested. For a dataset of 2847 proteins with domain/domains, the identification performance in this study was assessed by accuracy (Acc), which was defined as the proportion of amino acids correctly identified in the certain domain regions of the query sequences according to the information of protein structures in SCOP [40]. For example, suppose the domain regions of the query sequence have 100 amino acids; if 90 of which were correctly identified as belonging to domain regions while the other 10 were misjudged as sequence regions, then the Acc will be 90%. It was observed that when the community division method was based on edge betweenness, the Acc (*R*_C_*α*__) was stable at ~86% when *R*_C_*α*__ was around 5.0–7.5 Å, and Acc (*R*_C_*α*__) achieved the highest value of 86.68% when *R*_C_*α*__ was 5.0 Å. In addition, when the community division method was based on random walks, the Acc (*R*_C_*α*__) was ~81% when *R*_C_*α*__ was around 6.5–7.5 Å, and Acc (*R*_C_*α*__) achieved the highest value of 81.87% when *R*_C_*α*__ was 7.0 Å and the step size was 10. The identification performance proved that the optimal cutoff value for constructing the protein structure networks was 5.0 Å (C_*α*_-C_*α*_ distances), while the ideal community division method was community structure detection based on edge betweenness in this study. The results suggested that the amino acid interaction networks are an efficient method for describing the structure of proteins, and the different definitions of vertexes and edges do have important effect in this process.

## 2. Materials and Methods

### 2.1. Data Collection and Data Set Construction

The information on domains in proteins in this study were collected from ASTRAL SCOP [40] version 1.75 database. Protein domains in SCOP are grouped into species and hierarchically classified into families, superfamilies, folds, and classes [41]. This database organizes proteins hierarchically according to their families and folds, which is generally considered as the standard for protein structure classification [42]. In order to ensure the nonredundancy of the data, only these proteins with a pairwise sequence identity ≤30% were downloaded, and only those in which the structures were solved by X-ray crystallography with resolution ≤2.5 Å were kept for the clear structure of the proteins. Finally, the remaining 2847 proteins were left for this research. The compositions of the dataset were listed in Table 1.

### 2.2. Protein Structure Network

Protein structures can be represented as complex networks where amino acids are the nodes and their interactions are the edges [43]. In this study, each protein was considered a small self-governed network system. The structure of proteins was transformed into a complex network by taking amino acid residues as the vertexes and the interactions between the amino acid residues as edges. Various protein structure networks were constructed to investigate the protein structure and the influence of different strategies in building them.

**Figure 1:**
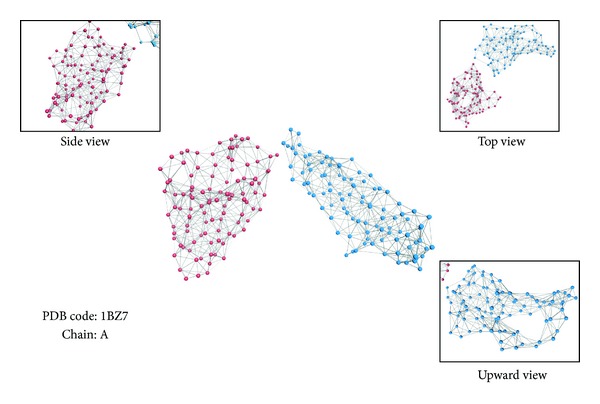
The amino acid residues interaction network. PDB code 1BZ7, chain A. The 3D structure of which is shown above together with its side, top and upward view. Here, the vertex is defined as C_*α*_, and the edge is C_*α*_-C_*α*_ distances which is set at 7.5 Å. Each point in the figure represents an amino acid in the chain, which is also the vertex of the network. Ligatures between the vertices are the edge of the network, which illustrate the interaction between the amino acids. For contrasting the figure of community division with this complex network, each vertex is colored based on its identity in SCOP. Here, reddish purple and blue represent different domain regions in this chain.

Here, edges are defined in three ways, and from which the optimal cutoff value was finally chosen. Two amino acid residues have a connection if (a) the distance between C_*α*_ (defined as *R*_C_*α*__) is 3–10 Å (step size of 0.5 Å, 15 different numerical values in all); (b) the distance between the centers of the side chains (defined as *R*_cent_) is 3–10 Å (step size of 0.5 Å, 15 different numerical values in all); (c) the distance between any atoms of the amino acid residues (defined as *R*_atm_) is 0–6 Å (step size of 0.5 Å, 13 different numerical values in all). The semidiameters of the atoms were taken into consideration. The amino acid residues interaction networks defined in this study are as shown in Figure 1, 3D structure of which is quite distinct.

### 2.3. Community Division

Tools for network analysis are firmly grounded on the results in graph theory [44], including which network community structure plays an important role in organizing and understanding the complex networks. The network communities were identified as dense groups of the network, whose nodes have a much stronger influence on each other than on the rest of the network [35]. Moreover, the connections between the residues are dense within domains, which express the community properties of such network well. Based on this characteristic, in this study, the community division methods were used to divide the whole sequences into potential domain regions. Two different methods were employed here: community structure detection based on edge betweenness and community structure via short random walks, and between which the more ideal one was finally choosen.

#### 2.3.1. Community Structure Detection Based on Edge Betweenness

Algorithms based on betweenness have been widely applied in various types of networks such as email messages, animal social networks, collaborations of jazz musicians, metabolic networks, and gene networks [33, 45–49]. For more detailed description of this method, refer to papers [45, 50]. The principle of the community structure detection based on edge betweenness is that it seems that all the shortest paths from one module to another must traverse through the edges connecting separate modules, which have high edge betweenness in that case.

As a result, this algorithm is performed by calculating the edge betweenness of the graph and removing the edge with the highest edge betweenness score gradually in order to obtain a hierarchical map. This rooted tree is the dendrogram of the graph, the leaves are the individual vertices, and the roots represent the whole graph. Finally, a numeric matrix is constructed using this algorithm.

#### 2.3.2. Community Structure via Short Random Walks

Algorithms based on random walks have been applied in various researches of networks [50, 51]. This algorithm tries to find densely connected subgraphs which are also known as communities in a graph via short random walks. The principle of this algorithm is that short random walks are likely to stay in the same community. It takes every single node as an independent community at first, then those of which tally with certain rules were incorporated together step by step. It introduces *r* as a distance between the vertices, which shall be small if the two vertices are in the same community and large if they are not.

## 3. Results and Discussion

### 3.1. Community Division Based on Edge Betweenness

**Figure 2:**
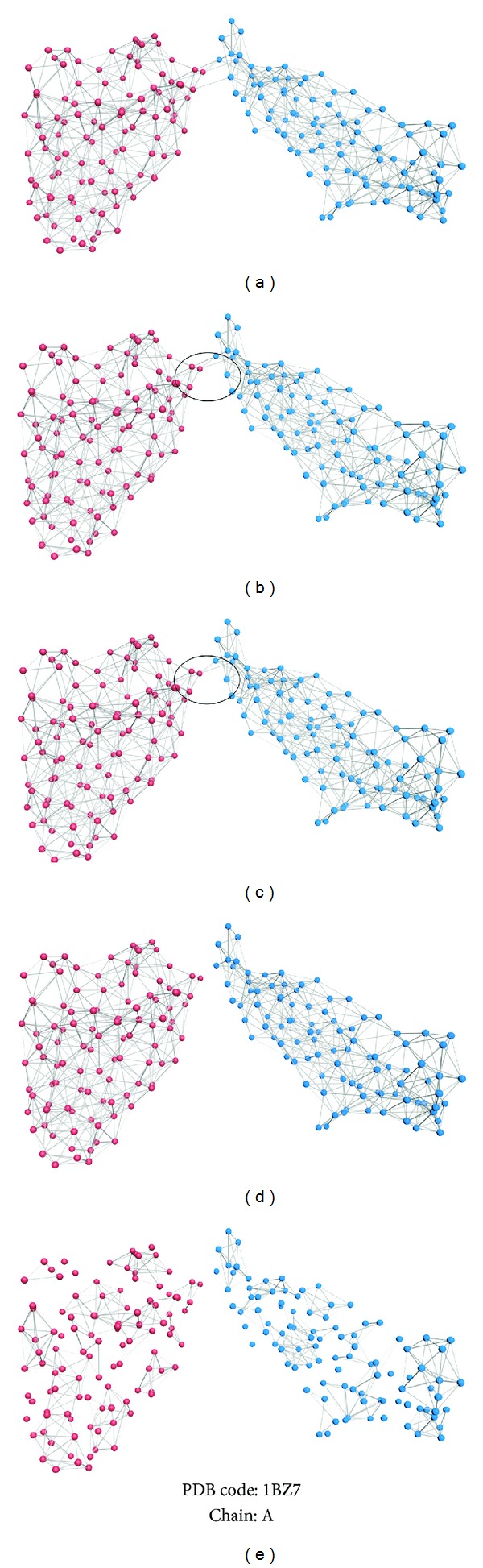
The flowchart of the amino acid interaction network together with community division method. PDB code 1BZ7, chain A. Each point in the figure represents an amino acid in the chain, which is also the vertex of the network. Ligatures between the vertices are the edge of the network, which illustrate the interaction between the amino acids. Here, the reddish purple and blue represent different domain regions in this chain based on the identity in SCOP. Firstly, an amino acid complex network was constructed with the vertex defined as C_*α*_, and the edge as C_*α*_-C_*α*_ distance which was set at 7.5 Å, as shown in (a). Secondly, community division was based on edge betweenness, and the first edge with the highest edge betweenness score was removed, as shown in (b). Thirdly, more edges were removed based on the algorithm, and (c) shows that three edges were removed. Fourthly, the community division was finished when the correct number of edges was removed, as shown in (d); two different domains have been clearly separated, and five edges were removed for this protein. Finally, if the community division is taken continually, more communities will be found in the complex network. (e) shows the result of community division for chain A of protein 1BZ7 after removing 500 edges in this complex network, and many more communities illustrate the wrong results according to the identity in SCOP.

In this section, community division method based on edge betweenness was applied on complex networks, and the effect of different cutoff values of edges for constructing complex networks was analyzed. Then, an optimized cutoff value was identified. The flowchart of these two steps, amino acid interaction network together with community division methods, is shown in Figure 2.

For the fairness of the contrast, all complex networks constructed by different cutoff values were analyzed by community division method, which insures the most optimal results. In order to obtain the best prediction performance, different cutoff values were evaluated based on multidomain proteins. 15 different values (3–10 Å) of the *R*_C_*α*__ and the *R*_cent_ (step size of 0.5 Å) were optimized, respectively, and so were other 13 different distance values (0–6 Å) of *R*_atom_ (step size of 0.5 Å). 

First, threshold of 7 Å, which has been reported to be an important distance parameter because all contacts are complete and legitimate (not occluded) at this distance [23], was analyzed. The results were obtained after the community division. The identification performance in this study was assessed by accuracy, which was defined as the proportion of amino acids correctly identified in the certain domain regions of the query sequences. When the *R*_C_*α*__ and the *R*_cent_ were 7 Å, respectively, the results are 86.21% and 85.16%, respectively. 

More cutoff values were tested via different strategies of vertex. First, the average accuracies for all the proteins defined by *R*_C_*α*__ were listed in Table 2. The results indicated that when the method was based on the edge betweenness, Acc (*R*_C_*α*__) achieved the highest 86.68% when *R*_C_*α*__ was 5.0 Å. When *R*_C_*α*__ was around 5.0–7.5 Å, the accuracies were around 86%, and the bias of the numerical values in this area was small (~1%). This illustrated that the cutoff values in this area reflected protein structure well. Second, the average accuracies for all the proteins defined by *R*_cent_ were listed in Table 3. The results indicated that Acc (*R*_cent_) achieved the highest 85.52% when *R*_cent_ was 7.5 Å. When *R*_cent_ was around 6.5–8.0 Å, Acc (*R*_cent_) showed relatively ideal values around 85%, which illustrated that the cutoff values in this area reflected protein structure well. However, the bias of the numerical values was evident for all the numerical values of *R*_cent_. Acc (*R*_cent_) were lower than 10% when *R*_cent_ was around 3.0–4.5 Å, which were generated by the otherness of the size of side chains. Third, the average accuracies for all the proteins defined by *R*_atom_ were listed in Table 4. The results indicated that when the distance between any atoms of the amino acid residues defined as *R*_atom_ was taken into consideration, the superiority of the diversity of the volume of atoms should also be taken into consideration. Acc(*R*_atom_) achieved the highest value of 85.59% when *R*_atom_ was 1.5Å. When *R*_atom_ was around 0.0–2.0 Å, Acc (*R*_atom_) showed relatively ideal values around 85%, and the bias of the numerical values in this area was small (~0.6%). When the cutoff values were bigger than 2.0 Å, Acc (*R*_atom_) decreased monotonically as *R*_atom_ increased. That is, overlarge *R*_atom_ will lead to the incorrect identification of the interactions among amino acids, which will distort the actual protein structure.

It was observed that when the community division method was based on edge betweenness, the Acc (*R*_C_*α*__) was stable at ~86%, which illustrated that the network characterization of protein structure would not be limited by its type. Furthermore, Acc (*R*_cent_) was ~1% lower than that of Acc (*R*_C_*α*__), which was generated by the cutoff value. That is, the side chains of the amino acids have a certain space volume, and a big cutoff value signifies the space overlap of the atoms from different amino acids, which is obviously inappropriate for protein structure. In conclusion, Acc (*R*_cent_) was lower than Acc (*R*_C_*α*__) and Acc (*R*_atom_), which illustrated that the space specificity of the side chains of amino acids affects the construction of the amino acids complex networks. It was observed that the highest accuracy obtained was 86.68% (*R*_C_*α*__ = 5.0 Å). That is, the optimal cutoff value was 5.0 Å (C_*α*_-C_*α*_ distances) when the ideal community division method was based on edge betweenness.

### 3.2. Community Division Based on Random Walks

In this section, the community division method based on random walks was analyzed. The same cutoff values were evaluated here based on multidomain proteins, that is, 15 different numerical values (3–10 Å) of the *R*_C_*α*__ and the *R*_cent_ (step size of 0.5 Å) and other 13 different numerical values (0–6 Å) of *R*_atom_ (using a step size of 0.5 Å). In addition, the step sizes of the community division based on random walks were also optimized here.

First, threshold of 7 Å [23] was analyzed for all the proteins. When the *R*_C_*α*__ and the *R*_cent_ were 7 Å, respectively, the results are listed in Table 5.

It was observed that when the community division method was based on random walks under the threshold of 7 Å via different step sizes, the highest Acc (*R*_C_*α*__) and Acc (*R*_cent_) were 81.93% and 80.70%, respectively. The numeric values of them all were ~4% lower than that for edge betweenness, which was generated by the method itself. That is, the algorithm based on the random walks attempted to find a given length called step size, which is obviously inappropriate for domains of different sizes. In large domains, a short length will not project all the amino acids in the same community.

More cutoff values were tested via different strategies of vertex. First, the average accuracies for all the proteins defined by *R*_C_*α*__ were listed in Table 6. The results indicated that Acc (*R*_C_*α*__) achieved the highest 81.87% when *R*_C_*α*__ was 7.0 Å and the step size was 10. When *R*_C_*α*__ was around 6.5–7.5 Å, the accuracies were around 81%, and the bias of the numerical values in this area was small (~1%). This illustrated that the cutoff values in this area reflected protein structure well. However, the numeric of Acc (*R*_C_*α*__) was ~5% lower than that for edge betweenness. Second, the average accuracies for all the proteins defined by *R*_cent_ were listed in Table 7. The results indicated that Acc (*R*_cent_) achieved the highest value of 80.77% when *R*_cent_ was 8.0 Å and the step size was 10. When *R*_cent_ was around 7.0–8.5 Å, Acc (*R*_cent_) showed relatively ideal values around 80%, which illustrated that the cutoff values in this area reflected protein structure well. However, the bias of the numerical values was evident for all the numerical values of *R*_cent_, which were generated by the otherness of the side chains. The numeric of Acc (*R*_cent_) was ~5% lower than that for edge betweenness, and Acc (*R*_cent_) was as low as 0% when *R*_C_*α*__ was around 3.0–5 Å, which may be produced by the looseness of the complex networks constructed under these thresholds. Third, the average accuracifes for all the proteins defined by *R*_atom_ were listed in Table 8. The results indicated that when the distance between any atoms of the amino acid residues defined as *R*_atom_ was taken into consideration, the superiority of the diversity of the volume of atoms should also be taken into consideration. Acc (*R*_atom_) achieved the highest value of 80.82% when *R*_atom_ was 1.0 Å and the step size was 10. When *R*_atom_ was around 0.0–2.5 Å, Acc (*R*_atom_) showed relatively ideal values around 80%, and the bias of the numerical values in this area was small (~1%). However, the numeric of Acc (*R*_atom_) was 5% lower than that for edge betweenness.

In conclusion, Acc (*R*_cent_) was lower than Acc (*R*_C_*α*__) and Acc (*R*_atom_). It was observed that when the community division method was based on random walks, the numeric of the accuracy was lower than that based on edge betweenness all the while, which indicated that the ideal community division method for this research was community structure detection based on edge betweenness. Moreover, the value of Acc (*R*_cent_) was the worst via both the two community division methods all along. Similar results were obtained in the study of side chain contact models; three models were compared and the isotropic sphere side chain (ISS) model was the worst in accuracy. They proved that the model which took the spatially anisotropic nature of the side chain into consideration would eliminate about 95% of the incorrectly counted contact pairs in the ISS model [26]. However, this kind of practical models do have less moderate computational cost than the popular representation model such as the use of C_*α*_ atom, which is proved to be effective for the kind of the data in this study. 

### 3.3. The Stability Analysis of the Method

To verify the stability of the method, 8 datasets were constructed based on multidomain proteins. The first dataset was composed of 100 proteins, and every other dataset contained 100 proteins more than the previous one. That is, the 8th dataset contained 800 proteins.

The same operations were taken based on these 8 datasets. Different numerical values of *R*_C_*α*__ (3–10 Å), *R*_cent_ (3–10 Å), and *R*_atom_ (0–6 Å) were optimized based on two community division methods. The highest accuracies for each dataset were listed in Tables 9 and 10.

It was observed that when the community division method was based on edge betweenness, Acc (*R*_C_*α*__) for each database got the highest results around ~86%–89% when *R*_C_*α*__ was ~5.00–5.50 Å, which were quite close to the result 86.68% when *R*_C_*α*__ was 5.00 Å. However, results for database one was a little bit different, 84.67% when *R*_C_*α*__ was 7.00 Å, which may be generated by the lack of statistically significant result in the small amount of the proteins. Acc (*R*_cent_) for each database got the highest results around ~85%-86% when *R*_cent_ was 7.50 Å, which were quite close to the result 85.52% when *R*_cent_ was 7.50 Å. However, results for database one was a little bit different, 82.51% when *R*_cent_ was 6.50 Å, which may be generated by the lack of statistically significant result in the small amount of the proteins. Acc (*R*_atom_) for each database got the highest results around ~82%–87% when *R*_atom_ was ~0.50−1.50 Å, which were quite close to the result 85.59% when *R*_C_*α*__ was 1.50 Å.

When the community division method was based on random walks, Acc (*R*_C_*α*__) for each database got the highest results around ~81%–85% when *R*_C_*α*__ was ~7.00–7.50 Å and the step size was 10, which were quite close to the result 81.87% when *R*_C_*α*__ was 7.0 Å and the step size was 10. Acc (*R*_cent_) for each database got the highest results around ~80%–84% when *R*_cent_ was 7.00–8.00 Å, which were quite close to the result 80.77% when *R*_cent_ was 8.0 Å and the step size was 10. Acc (*R*_atom_) for each database got the highest results around ~80%–84% when *R*_atom_ was ~0.50–1.50 Å and the step size was 10, which were quite close to the result 80.82% when *R*_C_*α*__ was 1.00 Å and the step size was 10. However, results for database one was a little bit different under these three conditions, which may be generated by the lack of statistically significant result in the small amount of the proteins.

It is observed from the results that the complex networks together with the community division methods constructed in this study were stable, which proved the creditability of the research. On the other hand, it was observed that when the community division method was based on edge betweenness, the Acc (*R*_C_*α*__) was stable at ~86% when *R*_C_*α*__ was around 5.0–7.5 Å, and the optimal cutoff value for constructing the protein structure networks was 5.0 Å (C_*α*_-C_*α*_ distances) in this study.

## 4. Conclusion

The main objective of this study is to explore the contribution of complex network together with its different definitions of vertexes and edges to describing the structure of proteins. When applying our method on a dataset of 2847 proteins with domain/domains, it was observed that when the community division method was based on random walks, the numeric of the accuracy was lower than that based on edge betweenness all the while, which indicated that the ideal community division method for this research was community structure detection based on edge betweenness. When the community division method was based on edge betweenness, the Acc (*R*_C_*α*__) was stable at ~86% when *R*_C_*α*__ was around 5.0–7.5 Å, and Acc (*R*_C_*α*__) achieved the highest value of 86.68% when *R*_C_*α*__ was 5.0 Å. The identification performance proved that the optimal cutoff value for constructing the protein structure networks was 5.0 Å (C_*α*_-C_*α*_ distances), while the ideal community division method was community structure detection based on edge betweenness in this study. The results suggested that the amino acid interaction networks are an efficient method for describing the structure of proteins, and the different definitions of vertexes and edges do have important effect in this process. Distance should be taken into consideration to prevent unnecessary deviation. Moreover, the optimized network model could be further applied in future study for the number and position of protein domain prediction.

## Figures and Tables

**Table 1:** The composition of proteins contained in the dataset.

Number of domains	1	2	3	4	5	6	7
Number of proteins	1450	1077	230	66	19	3	2

**Table 2:** The accuracies of all proteins defined by *R*_C_*α*__ based on edge betweenness.

Threshold	Accuracy
3 Å	2.15
3.5 Å	2.17
4 Å	78.96
4.5 Å	83.42
5 Å	86.68
5.5 Å	86.45
6 Å	85.54
6.5 Å	85.76
7 Å	86.21
7.5 Å	85.92
8 Å	85.21
8.5 Å	84.75
9 Å	84.28
9.5 Å	83.71
10 Å	83.86

**Table 3:** The accuracies of all proteins defined by *R*_cent_ based on edge betweenness.

Threshold	Accuracy
3 Å	2.14
3.5 Å	2.59
4 Å	3.79
4.5 Å	7.42
5 Å	33.99
5.5 Å	78.87
6 Å	84.53
6.5 Å	85.04
7 Å	85.16
7.5 Å	85.52
8 Å	84.89
8.5 Å	84.48
9 Å	83.83
9.5 Å	83.56
10 Å	83.40

**Table 4:** The accuracies of all proteins defined by *R*_atom_ based on edge betweenness.

Threshold	Accuracy
0 Å	85.06
0.5 Å	85.36
1.0 Å	85.58
1.5 Å	85.59
2 Å	85.06
2.5 Å	84.39
3 Å	83.73
3.5 Å	83.50
4 Å	83.95
4.5 Å	83.93
5 Å	83.51
5.5 Å	83.45
6 Å	83.31

**Table 5:** Acc (*R*_C_*α*__) and Acc (*R*_cent_) of all proteins based on random walks under 7 Å of different step sizes.

Step size	3	4	5	6	7	8	9	10
Acc (*R*_C_*α*__)	77.37	78.56	79.84	80.21	80.93	81.23	81.43	81.93
Acc (*R*_cent_)	76.39	77.62	78.56	79.12	79.64	80.05	80.13	80.70

**Table 6:** The accuracies of all proteins defined by *R*_C_*α*__ based on random walks.

Threshold	Accuracy
3 Å	0
3.5 Å	0
4 Å	67.14
4.5 Å	69.65
5 Å	73.84
5.5 Å	79.87
6 Å	80.39
6.5 Å	81.09
7 Å	81.93
7.5 Å	81.85
8 Å	80.97
8.5 Å	80.48
9 Å	80.46
9.5 Å	79.95
10 Å	79.71

**Table 7:** The accuracies of all proteins defined by *R*_cent_ based on random walks.

Threshold	Accuracy
3 Å	0
3.5 Å	0
4 Å	0
4.5 Å	0
5 Å	0
5.5 Å	5.05
6 Å	59.20
6.5 Å	78.34
7 Å	80.63
7.5 Å	80.63
8 Å	80.77
8.5 Å	80.20
9 Å	79.60
9.5 Å	79.64
10 Å	79.41

**Table 8:** The accuracies of all proteins defined by *R*_atom_ based on random walks.

Threshold	Accuracy
0 Å	80.39
0.5 Å	80.58
1.0 Å	80.82
1.5 Å	80.70
2 Å	80.79
2.5 Å	80.08
3 Å	79.55
3.5 Å	79.35
4 Å	79.24
4.5 Å	78.98
5 Å	78.68
5.5 Å	78.36
6 Å	77.49

**Table 9:** The optimal accuracies of each dataset based on edge betweenness.

Dataset	1	2	3	4	5	6	7	8
*R*_C_*α*__	7.00 Å	5.50 Å	5.50 Å	5.00 Å	5.50 Å	5.00 Å	5.50 Å	5.50 Å
Accuracy	84.67	89.08	87.07	86.52	87.35	87.26	86.95	86.50

*R*_cent_	6.50 Å	7.50 Å	7.50 Å	7.50 Å	7.50 Å	7.50 Å	7.50 Å	7.50 Å
Accuracy	82.51	86.93	86.50	85.74	86.17	86.58	85.85	85.49

*R*_atom_	1.00 Å	1.00 Å	0.50 Å	1.00 Å	1.50 Å	1.00 Å	1.00 Å	1.00 Å
Accuracy	82.89	87.54	86.24	86.13	86.94	86.61	85.61	85.80

**Table 10:** The optimal accuracies of each dataset based on random walks.

Dataset	1	2	3	4	5	6	7	8
*R*_C_*α*__	6.00 Å	7.50 Å	7.50 Å	7.50 Å	7.50 Å	7.00 Å	7.50 Å	7.00 Å
Step size	10	10	10	10	10	10	10	10
Accuracy	75.34	85.00	82.46	81.61	83.20	83.39	82.25	81.93

*R*_cent_	7.00 Å	7.00 Å	8.00 Å	8.00 Å	8.00 Å	8.00 Å	7.50 Å	7.00 Å
Step size	10	10	10	10	9	10	10	10
Accuracy	74.62	84.95	80.97	80.89	81.84	82.67	80.61	80.79

*R*_atom_	0.50 Å	1.50 Å	0.50 Å	1.00 Å	1.50 Å	1.00 Å	1.00 Å	1.00 Å
Step size	10	10	10	9	10	10	10	10
Accuracy	74.85	84.66	81.20	81.11	82.36	82.97	81.45	80.95
